# Evaluation of the “Eat Better Feel Better” Cooking Programme to Tackle Barriers to Healthy Eating

**DOI:** 10.3390/ijerph14040380

**Published:** 2017-04-04

**Authors:** Ada L. Garcia, Rebecca Reardon, Elizabeth Hammond, Alison Parrett, Anne Gebbie-Diben

**Affiliations:** 1 Human Nutrition, School of Medicine, College of Medical, Veterinary & Life Sciences University of Glasgow, Glasgow G31 2ER, UK; rreardon91@gmail.com (R.R.); Alison.Parrett@glasgow.ac.uk (A.P.); 2 Public Health Directorate, NHS Greater Glasgow and Clyde, Glasgow G12 0XH, UK; Elizabeth.Hammond@ggc.scot.nhs.uk (E.H.); Anne.Gebbie-Diben@ggc.scot.nhs.uk (A.G.-D.)

**Keywords:** diet behaviour, socioeconomic deprivation, community-based intervention, food waste

## Abstract

We evaluated a 6-week community-based cooking programme, “Eat Better Feel Better”, aimed at tackling barriers to cooking and healthy eating using a single-group repeated measures design. 117 participants enrolled, 62 completed baseline and post-intervention questionnaires, and 17 completed these and a 3–4 months follow-up questionnaire. Most participants were female, >45 years, and socioeconomically deprived. Confidence constructs changed positively from baseline to post-intervention (medians, scale 1 “not confident” to 7 “very confident”): “cooking using raw ingredients” (4, 6 *p* < 0.003), “following simple recipe” (5, 6 *p* = 0.003), “planning meals before shopping” (4, 5 *p* = <0.001), “shopping on a budget (4, 5 *p* = 0.044), “shopping healthier food” (4, 5 *p* = 0.007), “cooking new foods” (3, 5 *p* < 0.001), “cooking healthier foods” (4, 5 *p* = 0.001), “storing foods safely” (5, 6 *p* = 0.002); “using leftovers” (4, 5 *p* = 0.002), “cooking raw chicken” (5, 6 *p* = 0.021), and “reading food labels” (4, 5 *p* < 0.001). “Microwaving ready-meals” decreased 46% to 39% (*p* = 0.132). “Preparing meals from scratch” increased 48% to 59% (*p* = 0.071). Knowledge about correct portion sizes increased 47% to 74% (*p* = 0.002). Spending on ready-meals/week decreased. Follow-up telephone interviewees (*n* = 42) reported developing healthier eating patterns, spending less money/wasting less food, and preparing more meals/snacks from raw ingredients. The programme had positive effects on participants’ cooking skills confidence, helped manage time, and reduced barriers of cost, waste, and knowledge.

## 1. Introduction

Poor diet is a major public health challenge associated with high rates of obesity and increased risk of chronic disease [1,2,3]. In Western countries like Scotland, poor diets are low in fruit and vegetables, fibre rich foods, and oily fish, and are high in energy, red meat, saturated fats, salts, and sugars due to a high intake of confectionary and processed foods, as well as ready to eat meals and takeaways [4]. Poor diet is linked to low levels of education, low income, and high socio-economic deprivation [4,5,6,7]. Insufficient food knowledge and practical cooking skills are further contributing factors to poor diet [8,9]. Additionally, busy schedules, competing priorities, daily stressors, and financial constraints are other barriers to healthy home cooking. These barriers encourage reliance on cheap pre-packaged, processed, and ready-to-eat meals [9,10,11,12].

Creating and supporting healthier food environments and consumption patterns using a health promotion approach is therefore needed to tackle poor diet [13]. This is of particular importance for those living in socio-economic deprivation because they require increased “support, education, and skills development” to overcome barriers such as “food affordability and availability, and lack of food skills” [14]. The government recommends overcoming these barriers by engaging in “practical and achievable steps, supplemented by the promotion of healthy food choices and meal preparations, to help the public take steps in moving towards a healthier diet” [14].

Cooking skills programmes have been used as a strategy to improve and promote cooking confidence, healthier food choices, and well-being [9,10,11,15,16]. They are attractive to health practitioners because they allow the delivery of nutrition education via inclusive social activities [11]. In Scotland, cooking programmes are widely used in different settings and are delivered by the third sector or the National Health Service (NHS) [15]. However, cooking courses vary greatly in their organisation, content, and delivery and they often lack an evaluation framework [17]. Thus, developing a programme which targets the main barriers to healthy eating, offers an evaluation scheme, and that can be used as a reference to others is a promising strategy for diet improvement. Up to now, no other programme has specifically aimed to tackle time, cost, and waste. The aim of this study was to evaluate the effectiveness of a community-based cooking skills programme developed by the NHS Greater Glasgow and Clyde in Scotland (NHSGGC) in increasing confidence in cooking and tackling the barriers of time, cost, waste, and knowledge of cooking and healthy eating in the short (post-intervention) and mid-term (3–4 months follow-up).

## 2. Materials and Methods

### 2.1. Study Design and Ethics

The evaluation used a single group pre-test, post-test, and follow-up test repeated measures design with both quantitative and qualitative elements. The baseline and post intervention evaluation components were part of a service evaluation. This study was conducted in accordance with the guidelines laid down in the Declaration of Helsinki and all procedures involving human subjects were approved by the Medical, Veterinary and Life Sciences (MVLS) Ethics Committee (200140157).

### 2.2. Programme Setting

The cooking programme was developed by NHSGGC Public Health, Health Improvement Directorate. The purpose was to deliver cooking classes to improve cooking confidence, skills, and nutrition knowledge to tackle poor dietary behaviours. A novel aspect was to target the previously identified barriers to cooking and healthy eating: time, cost, and waste [18]. The programme incorporated elements of food planning, purchasing, preparation, cooking from scratch (using raw or basic ingredients), and storage. The target population was comprised of vulnerable groups living in areas of socioeconomic deprivation in Glasgow such as pregnant women, older adults, overweight and obese individuals, carers of young children (early years), and those with long-term health conditions. The programme was piloted before being implemented (data not shown here) and a quality assurance and evaluation framework was developed using a similar evaluation design as previously reported [16]. Fifteen community centres were invited to commission the delivery of the programme along with certified community-trained chefs. The course was branded the “Eat Better Feel Better cooking skills course” to align the programme to the Scottish Government campaign “Eat Better Feel Better” [19].

### 2.3. Recruitment and Delivery of the Cooking Skills Programme

Recruitment to the programme was facilitated by the different centres. The programme was implemented from January to March 2015, with community-trained chefs delivering a total of 15 6-week courses, and appropriately tailoring the course to each target group. Each course session took place once a week for approximately two-hours and consisted of a specific practical cookery component, along with an educational element which comprised of healthy eating messages and activities, based on that week’s lesson plan and learning outcomes. The core healthy eating messages were based on the Eat Well Plate, traffic light system, how to get five portions of fruit and vegetables a day, preparing healthy breakfasts, healthy packed lunches, and takeaways. The core themes of the programme were consistent across the different target groups and included food planning, purchasing, preparation, cooking, and storage. An array of additional resources were provided to aid in the delivery of each session when working with specific target groups. The full programme can be freely accessed at http://www.nhsggc.org.uk/about-us/professional-support-sites/community-cooking-network/ while the evaluation forms are provided in Supplementary Material 1.

### 2.4. Evaluation Methodology and Questionnaires

Baseline intervention and post-intervention questionnaires were part of the service evaluation and were used to collect information to evaluate the immediate programme effects. Baseline intervention questionnaires were completed by the participants and collected by the chefs at the beginning of the first session, along with the participant’s written consent to the follow-up evaluation. The post-intervention questionnaires were completed by the participants and collected by the chefs at the end of the final session.

For the follow-up evaluation, those participants who consented to being contacted, and who provided a valid telephone number, were called by an independent researcher and invited to participate in a brief and simple telephone interview, with prior verbal consent. The interview consisted of 12 qualitative questions (Supplementary Material 2) about why they attended the course, their food shopping habits, snack and meal practices, and suggestions on how to improve the course. All participant responses were recorded directly onto individual questionnaires labelled with their identification code. All responses were entered into a Microsoft Word document and response frequencies appropriately themed into categories. At the end of the interview, participants were asked to complete a longer paper-based questionnaire with matching questions for comparisons with baseline and post-intervention questionnaires. Those who agreed were asked for a mailing address and an information package with pre-paid return envelopes were sent to them via the post.

The three written questionnaires consisted of the same core questions. The questionnaires were developed at a lower literacy level because the clientele was expected to have a varied level of literacy. Several of the constructs were identical to those previously validated [20].

Fourteen core groups of questions were asked about several aspects such as cooking/preparation methods used, shopping and eating habits, knowledge on portion sizes and nutrient content, eating behavior, and confidence on cooking skills. Core questions could be answered using either yes/no, ticking specific boxes or were open ended questions. Three core questions used Likert scales as appropriate to the question. For the Likert-style scales, confidence was rated on a scale of 1 (not at all confident) to 7 (very confident) and included 12 questions; food frequency intake was rated on a scale of “never” to “more than once a day” and included four questions; and food practices was rated on a scale of “never” to “always” and included 9 questions. Six extra qualitative questions were added to the post-intervention questionnaire to solicit information with respect to: enjoyment and/or difficulties with the programme; any cooking attempts at home; and to obtain general feedback.

### 2.5. Statistical Analysis

All statistical analyses were done using the IBM SPSS Statistics software package (version 21.0, IBM, Foster City, CA, USA). Intervention effects between baseline and post intervention were tested using the Wilcoxon signed-rank test for scales and Friedman’s Analysis of Variance (ANOVA) for continuous data. Similar tests were used for comparisons between baseline and follow-up. Statistical significance was accepted at *p* < 0.05.

Cronbach’s α was used to determine internal consistency of 12 items for testing confidence (i.e., “following a simple recipe”, “storing foods safely”, etc.) using Likert-style scale questions in all three questionnaires in the same population. At baseline (*n* = 117; 80 valid), post-intervention (*n* = 68; 59 valid), and follow-up (*n* = 23; 21 valid), the Cronbach’s α values for the 12 confidence items were 0.912, 0.904, and 0.945, respectively, showing high reliability.

## 3. Results

**Figure 1:**
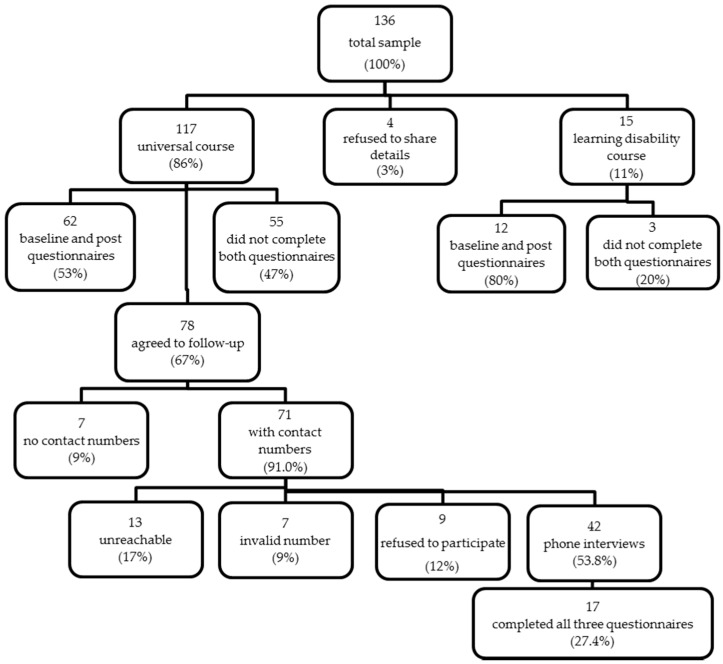
Eat Better Feel Better cooking skills programme participation and questionnaire completion rate.

A total of 136 subjects participated in one of the 15 courses delivered in the council areas of Glasgow City, Inverclyde, Renfrewshire and East Renfrewshire. From this, four refused to share details and 15 had learning disabilities and were excluded from the analysis, leaving 117 participants to be included in this study as shown in Figure 1.

From the 117 participants, 62 completed both the baseline and post-intervention evaluation questionnaires (Table 1). The majority of participants were female, over 45 years old, and of Scottish descent. Most attended five-to-six sessions, were from Inverclyde, lived independently or with one other person, and fell within the first and second Scottish Index of Multiple Deprivation (SIMD) quintiles (the areas of highest deprivation).

Two-thirds (*n* = 78) of participants agreed to be contacted for the follow-up evaluation. Forty-two were available and agreed to complete both the telephone interview and follow-up questionnaire, but only 17 completed all three-time-point evaluations. The demographics for the follow-up group were similar to the baseline and post-intervention group for the number of sessions attended, gender, ethnic background, and SIMD quintiles, but this group comprised of a much higher proportion of participants over 45 years of age and from households of 1–2 people. Although several demographic characteristics were similar between the groups, the follow-up group was not completely representative of the baseline/post-intervention group. Due to this, results of the follow- up findings will be presented for comparisons between baseline and follow-up.

### 3.1. Effects between Baseline and Post-Intervention (n = 62)

Meal preparation techniques improved between baseline and post-intervention with participants reporting a reduction in “putting ready meals in microwave” (46% vs. 39%), “putting together ready-made ingredients to make a meal” (52% vs. 46%), and an increase in “preparing meals from scratch” (48% vs. 59%), and a slight change in reporting “Don’t cook at all” (3% vs. 5%). However, these changes were not statistically significant (*p* > 0.05).

The effects of the programme on cooking time were evaluated by the frequency in “planning what to cook before shopping” which did not change between baseline and post-intervention, whereas participants reported a significant change in median response (*p* = 0.039) in the frequency in “cooking in bulk” from “rarely/sometimes” (median = 2) at baseline to “sometimes” (median = 3) at post-intervention (Table 2).

Programme effects on food cost showed that the median values for self-reported spending on food per week were identical between baseline and post-intervention (£40.00, *p* = 0.249). The median values for the amount of money reported on takeaway/fast food per week at baseline and post-intervention were £10.00 and £8.00, respectively. Food waste effects were reported as frequencies for “throwing away leftover food” which did not change between baseline and post-intervention (Table 2).

Eating behaviours between baseline and post-intervention showed that median values for behaviours such as eating breakfast, snacking between meals, and eating meals at regular times did not change (Table 2). However, when assessing food frequency consumption, an effect between baseline and post-intervention was observed for leftover foods (*p* = 0.022) and using pre-prepared foods (*p* = 0.032) (Table 3). These changes were not evident when reporting median values, but when looking at the frequencies of intake (detailed data not shown), the statistical significance is explained. There was a positive shift in participants reporting eating more leftover foods from “never” to “once a week” at baseline (88%) to “2–4 times a week” to “once a day” at post-intervention (97%). Similarly, for pre-prepared food consumption there was an observable shift towards reporting lower consumption at post-intervention (26.2% at 2–4 times a day; 26.2% at less than once a week; 16.4% at never) vs. baseline (32.6% at 2–4 times a day; 19.4% at less than once a week; 11.5% at never). No significant changes were observed for the frequency of eating salad or fish between baseline and post-intervention.

Food and nutrition knowledge was tested objectively and subjectively in four aspects. First they were asked to answer whether they understood why eating a balanced diet was important by answering yes or no. At baseline, 86% answered yes to this question while 95% did at post-intervention, a small but not significant improvement (*p* = 0.059). Those who responded “yes” were asked to provide written responses as to why they thought eating a balanced diet is important. At both time points, the majority stated it was important “for good health”, followed by increased responses of “for weight management”, “for nutrition”, and “healthy body and lifestyle”.

Secondly, participants were asked to label the sugar content of six common breakfast cereals (cornflakes, plain porridge, coco pops, crunchy nut cornflakes, rice krispies, and weetabix) and the fat content of six common food products (plain scone, fresh fruit, standard bag of crisps, sausage roll, baked crisps, and vegetable soup) in the form of “low”, “medium”, or “high”. There were no significant changes in correct sugar content labeling between baseline and post-intervention (*p* ≥ 0.257) (Table 4). However, small increments in reported correct answers were observed at post-intervention for identifying high sugar containing cereals. There was a significant improvement observed between baseline (74%) and post-intervention (90%) of correct responses for sausage roll fat content (*p* = 0.011) (Table 5).

**Figure 2:**
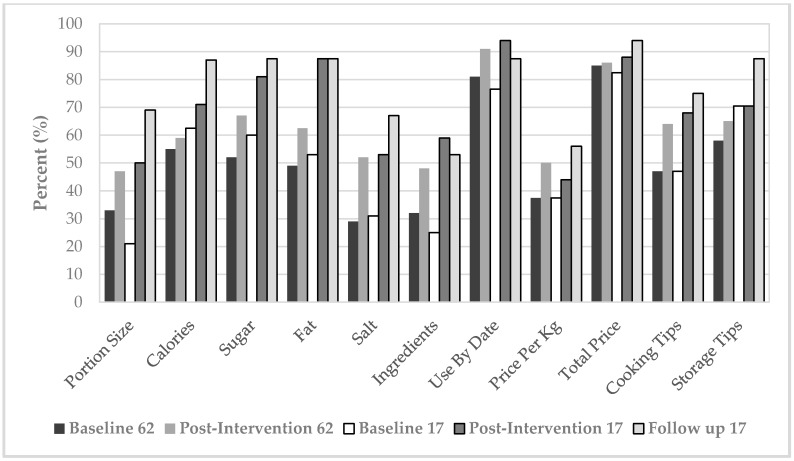
Food label elements checked at baseline and post-intervention (*n* = 62) and 3–4 months follow-up (*n* = 17).

Thirdly, participants were asked if they could identify correct portion sizes. There was a significant change detected between baseline and post-intervention (*p* = 0.002), with a 27% significant improvement in this knowledge from 47% to 74%, respectively (Figure 2).

The fourth aspect of knowledge was related to food labels. There was an overall positive increasing trend in objectively measured food label reading by the participants from baseline to post-intervention (Figure 2). Of the 11 food label items asked in the questionnaire, the label scores indicated a statistically significant increase in median food label reading between baseline and post-intervention (*p* = 0.038).

Cookery skills confidence was tested using twelve confidence elements and 11 of these showed statistically significant differences between baseline and post-intervention (*p* < 0.05) (Table 6). Significant median increases in confidence were observed for these elements relating to following recipes (5 vs. 6, *p* = 0.003), planning meals (4 vs. 5, *p* < 0.05), and using leftovers (4 vs. 5, *p* = 0.002); as well as cooking from raw ingredients (4 vs. 6, *p* = 0.003), storing food safely (5 vs. 6, *p* = 0.002), shopping on a budget (4 vs. 5, *p* = 0.044), and reading food labels (4 vs. 5, *p* < 0.001), from baseline to post-intervention, respectively.

### 3.2. Effects at Follow-Up (n = 17)

The small number of participants who completed all evaluation questionnaires (*n* = 17) showed that several aspects of the programme were sustained at 3–4 months after attending the programme in comparison with baseline values. Improvements in preparation and cooking time were observed between baseline and follow-up with less participants (59% at baseline vs. 23.5% at follow-up) reporting to “put ready meals in the microwave” and more participants (59% at baseline vs. 88% at follow-up) reporting to “prepare meals from scratch”. Other aspects such as frequency in “planning what to cook before shopping” increased from “sometimes” (median = 3) to “usually” (median = 4) from baseline to follow-up (*p* = 0.008) and “cooking in bulk” increased from “rarely/sometimes” (median = 2.5) to “sometimes (median = 3)” from baseline to follow-up (*p* = 0.038) (Table 2). For barriers related to food costs and waste, the follow-up results showed no difference in money spent on food (£40.00, *n* = 17, *p* = 0.858). However, a non-significant reduction in the amount of money spent on take-away meals was observed (£2.00, *p* ≥ 0.094).

Eating behaviours such as “eating breakfast” were similar in the follow-up group compared to baseline (Table 2). For specific food eating frequencies, at follow-up participants reported an increase median (median = 3) in “eating leftover foods” compared to baseline (media = 2), however this was not statistically significant (Table 3). Other frequencies in consumption of specific food groups showed small improvements. There were significant median increases observed in eating salad from “once a week” (median = 3) to “2–4 times a week” (median = 4, *p* = 0.024) and eating oily fish from “less than once a week” (median = 2) to “2–4 times a week” (median = 4, *p* = 0.039) between baseline and follow-up.

At follow-up, 94% vs. 70.6% at baseline responded yes to whether they understood why a balanced diet was important, which suggests a gain in this knowledge (*p* = 0.046). For specific questions on sugar and fat content labeling, non-significant differences between baseline and follow-up responses (*p* ≥ 0.326, *p* ≥ 0.549, respectively) were indicated (Table 4 and Table 5). However at follow-up there was an observable decline in correct responses for both sugar and fat content labeling compared to baseline (Table 4 and Table 5). For portion size knowledge, significant changes between baseline and follow-up (*p* = 0.004) were observed, with more participants (88%) reporting correct portion size comprehension at follow-up compared to baseline (35%). The positive increasing trend in objectively measured food label reading by the participants was sustained at follow-up (Figure 2).

Almost all confidence ratings for preparation and cooking practices were significantly increased at follow-up compared to baseline (*p* > 0.05). Improvements in “cooking using raw ingredients” and “cooking whole raw chicken” confidence were not statistically significant (Table 6).

### 3.3. Effect of Strength of Exposure

The effect of attendance to either 5–6 or less sessions was analyzed for food preparation, cooking and eating practices (Table S1 Supplementary Material 3), and cookery skill confidence (Table S2 Supplementary Material 3) for those participants who completed baseline and post-intervention (*n* = 62), but not for follow-up because of very low numbers. Higher attendance (*n* = 32) resulted in different results from those of all participants for just 2/9 constructs, “planning what to cook before shopping” reached significance (*p* = 0.006) for those who attended 5–6 sessions compared to those who did not, while “cooking in bulk” was no longer significant (*p* = 0.191). On the other hand, those participants who attended less sessions had positive results in 3/9 constructs when compared to all participants. Statistical significance was reached for “I think it is time consuming using raw ingredients when cooking” (*p* = 0.034), “I cook in bulk” (*p* = 0.039), and “I have snacks in between meals” (*p* = 0.046).

The results of cooking skill confidence showed no difference in any of the confidence constructs between those attending 5–6 sessions and the whole group but some of the confidence constructs in those attending less sessions did not reach statistical significance in 5/12 constructs, thus attending more sessions is more effective in increasing confidence and should be considered as an important requirement when planning and implementing cooking interventions.

### 3.4. Qualitative Evaluation at Post-Intervention (n = 62)

Sixty-two participants completed the qualitative section of the post-intervention evaluation. These were some of the themes and quotes from the questionnaires.

#### 3.4.1. Theme: Applying Cooking Skills, Nutrition Knowledge, and Eating Behavior Aspects

Many participants acknowledged that the educational aspects and learning experiences contributed very much to the enjoyment and value of the course. These included: learning about using and tasting new ingredients and recipes (*n* = 18); learning nutritional and cooking tips, as well as budgeting (*n* = 5). Over half (*n* = 34) of the participants said they have increased cooking new dishes and healthy meals from scratch at home, they are eating healthier food choices, and balancing meals while cutting back on unhealthy foods (*n* = 22). Other also said they continued trying new foods and recipes at home (*n* = 8), as well as reading food labels (*n* = 8) and using the traffic light system (*n* = 6). Participants’ quotes included: “*Trying different foods I’d never tasted and learning healthy cooking and eating isn’t as difficult as first thought*” (respondent 074); “*I found the course very knowledgeable and when I shop I look at the labels and try to follow the instructions*” (respondent 045).

#### 3.4.2. Theme: Confidence

A large number of participants (63%) reported that the course helped them by: improving their confidence in cooking and socializing with new people and learning new cooking tips. Participants expressed the following: “*It helped me a lot to build my confidence on cooking raw meat, and making the food a lot healthier, sort[ing] portion sizes out*” (respondent 063); *“More confident now when I am cooking” *(respondent 104).

#### 3.4.3. Theme: Savings and Budgeting

Some participants claimed improved cost/budget and time saving through the usage of leftovers, decreasing overall food waste, and planning/preparing more meals; however, these statements differ from the quantitative post-intervention results. Statements from participants included: “*I save on my shopping bill using leftovers*” (respondent 060); *“How easily you can make things from scratch and how cheaply you can make a meal”* (respondent 131).

#### 3.4.4. Theme: Socialization and Health Awareness

A further emerging theme was how the course supported and encouraged participants to meet, interact, and socialize with new people (*n* = 19). The company and socialization element of the course was expressed to be a reason why they found it enjoyable (*n* = 12). Another theme that arose included increased awareness of one’s own dietary behaviours, practices, and health. Participants expressed the following in relation to this theme: *“Getting out and meeting people and enjoying cooking with ones in class”* (respondent 083); *“[The course] helped to socialize with people and the course made me more confident in myself” *(respondent 043); *“Putting more thought into using what ingredients I have to make meals; not just going out and buying without checking the cupboard first”* (respondent 119); *“This course opened my eyes and really made me think about being a healthier person”* (respondent 075).

#### 3.4.5. Theme: Overall Course Feedback and Adaptations for Improvement

Participants were also asked to provide feedback on the course as-a-whole and adaptations for improvement and the majority (84%) responded that there were no difficulties with the programme while the rest mentioned that the duration of the sessions were too short, problems with reading the recipes, and tasting and touching certain foods. Furthermore, most (87%) answered “no” when asked if they found anything from the course too difficult to try at home. While many participants enjoyed and expressed favourable elements about the course, common statements and suggestions for improvement included increasing class size, extending course hours and duration, allowing for flexible class times, and having the course in a larger facility. The majority (64.5%) provided no response or reported no suggestions for improving the course. Quotes from this theme included the following: *“No real complaints—although sometimes we could have done with another hour!”* (respondent 047), *“It was not long enough I would like the course to go on for more weeks”* (respondent 083).

### 3.5. Follow-Up Telephone Interview (n = 42)

Forty-two out of the 78 participants who agreed to partake in the follow-up evaluation, completed the telephone interview (Figure 1). In response to “what made you come along to the course”, many revealed that they were interested in learning about healthy eating and cooking skills (*n* = 25), and were either referred or invited to participate (*n* = 13), while a few mentioned socializing with others (*n* = 4) and for health reasons (*n* = 3). Forty (95%) reported that the course was helpful to them and their family, because it enabled the household to cook and eat healthier and better foods (*n* = 18); to try new things (*n* = 7); to choose better options in the store (*n* = 6); to enhance cooking skills (*n* = 5); or enabled them to socialize more (*n* = 3).

Thirty-six participants (86%) reported that their shopping had changed as a result of the course. Shopping changes mentioned included: buying more fresh foods and healthier food options (*n* = 17); using the Traffic Light System and reading labels more (*n* = 10); buying less or no packaged meals/processed foods (*n* = 9); buying a larger variety of ingredients (*n* = 6); no impulse buying (*n* = 3); and looking for bargains/deals and budgeting (*n* = 3). Quotes from the interviews included the following: *“More fresh and healthy foods…less boxed and packaged meals”* (respondent 116), *“Changed cereals I buy as (I) found the ones I bought were too high in sugar”* (respondent 075).

In response to “are you spending less money on food shopping”, 24 (57%) reported “Yes”, and reported the reasons for this as: bulk cooking, using leftovers and wasting less food (*n* = 7); buying less processed/packaged goods and more fresh foods (*n* = 7); not buying brand items (*n* = 5); and changing to a different grocery store (*n* = 4). Those who reported “no” (*n* = 15, 36%) mentioned it was due to habit and because they already had healthy food options in place of packaged/processed foods. The participants provided comments such as: *“Yes, sometimes…shown how to budget…buying more fresh and use leftovers more for recipes”* (respondent 039) and *“Not sure…difficult to tell…changed things I buy, but same about…so [buying] better food products, more vegetables and more quality foods”* (respondent 021).

The majority (*n* = 37, 88%) reported preparing more meals and snacks from raw ingredients. Motives to do so included: eating healthier, more nutritious foods (*n* = 21); enjoying the cooking process (*n* = 9); food is much tastier (*n* = 7); to lose weight (*n* = 5); and that it is simple and quick to do (*n* = 5). Quotes for this theme were *“Cooking from scratch…have to be well prepared…but makes it easier for me during the week”* (respondent 135) and *“Enjoy cooking everything from scratch; the taste, more nutritious…I like smelling the foods”* (respondent 124).

As for snacks, 20 participants (48%) reported eating the same amount after the course because of never being a snack eater (*n* = 11), eating healthier snack options (*n* = 7), and habit (*n* = 5). Fifteen (36%) reported eating less snacks during the day because of better/proper meals eaten at regular times (*n* = 10), and eating more fruit as a snack option (*n* = 5). Most participants (*n* = 33, 79%) mentioned that they wasted less food by means of: freezing/storing leftovers properly (*n* = 17); using leftover meals to cook new meals (*n* = 17); cooking in bulk (*n* = 8); and cooking correct portion sizes for the day (*n* = 6). Expressions such as *“Using more fruit and vegetables…not going rotten as use more quickly…look at Use By Date…more aware/realize not to chuck it out yet”* (respondent 100) and *“… [with] old bananas, I make frozen goods or bread; make soups with more outdated vegetables…I buy smaller loaves of bread to reduce waste”* (respondent 119) were provided during the interviews.

Overall, the interview revealed that a vast majority benefited from the course. They reported that they had developed healthier eating patterns, were spending less money on food, were preparing more meals and snacks from raw ingredients, and were wasting less food.

## 4. Discussion

The programme was delivered with the aim of reducing the perceived barriers of time, cost, waste, and knowledge in cooking practice, while targeting vulnerable groups living in socioeconomic deprivation. The programme was successfully developed, piloted and delivered across different geographical areas in the central west region of Scotland and targeted to different vulnerable groups, thus the course can be proposed to be a reference programme for delivering cooking skills and improving nutrition knowledge across similar areas in Scotland.

The majority of participants attended 5–6 sessions and those who attended less or did not report attendance were fewer in number; this had an effect on confidence ratings for preparation and cooking practices between baseline and post-intervention, however it is important to consider that the number of participants who attended less sessions was very small, thus these results will need to be verified in another study with the main aim of testing the strength of exposure and with a higher number of participants purposely allocated to low vs. high exposure.

We aimed to reduce the perceived barrier of time as previously described as a constraint to cooking and to healthy eating [9,12]. There were significant self-reported improvements for preparing more meals from scratch, planning meals before shopping, and cooking in bulk from baseline to post-intervention, suggesting that the participants are using or managing their time more effectively and efficiently when it comes to food planning, preparation, and cooking. These improvements were corroborated by the follow-up telephone interview in which 88% of the participants reported that they cooked more meals from scratch because of the health and nutritional value. A number stated that they were doing less impulse buying, which suggests healthier food planning habits. The positive improvements observed in this study are consistent with results of the Jamie’s Ministry of Food cooking programme evaluation [10,21].

Food affordability and increase in food waste are further barriers to healthy eating [22,23]. According to the 2013 Living Costs and Food Survey, United Kingdom (UK) households were spending on average £42.18 per person per week [24]. In this study, the median self-reported expenditures on food/drink (£40.00) and takeaway/fast food (£8.00) at baseline and at follow-up, £40 and £2 respectively, may fall below the national value since findings were not reported per person basis, and according to the demographic data in our study most participants lived alone or with another person. According to this self-reported food spending there were no changes in overall food expenditure across the different time points but just a reduction in money spent on takeaway/fast food. Nevertheless, positive practices such as using more leftover foods could result in reducing food waste and more value for the money spent on food. Additionally, the knowledge of cooking in bulk, including proper food storage, is consistent with wasting less and monetary savings, and was corroborated by the telephone interviews, with 79% stating that they are wasting less food due to properly freezing and storing leftovers and using them more in new meals. The waste reduction aspect has not been explored by other cooking programmes and is an important novel element to consider in future planning of similar interventions. This is important in a larger context because it has been well documented that food waste is a large problem in the UK [25]. Learning how to manage food waste might have implications for reduction in household budget, but also for sustainability and environmental reasons [26].

The telephone interviews (*n* = 42, 53% response rate) provided further insight into shopping patterns and spending amounts and confirmed the findings of the questionnaires. Eighty-six percent acknowledged that their shopping had changed since the programme started, with circa 60% of participants indicating they spent less money on food. The main reasons provided were buying less packaged/process meals and more fresh food, which is similar to previous reports [10,16]. Participants also indicated the use of leftovers, waste less, not buying brand items, seeking deals, and changing to a different store. A further important perceived positive change to spending patterns was a substantial decline in takeaway/fast food spending per week by £6.00 at follow-up, which suggests improved changes in food shopping patterns as a result of preparing more meals from scratch [10,16]. These results have important implications for those households with lower incomes as it shows an improvement in the diet quality.

An important aspect of the course was increased basic nutrition knowledge. The course achieved improvements in participant’s practical use of food label components; understanding correct portion sizes and awareness of the importance of a balanced diet for health. This gain in knowledge suggests that participants acquire skills for making healthier food choices. Better understanding of correct portion size may indicate more deliberate and controlled food intake, which is essential in regulating over-eating and managing food waste well, which in turn influences food spending. The findings are further supported by responses in the telephone interviews—i.e., continued use of the Traffic Light System/food labels, conscious buying, and purchasing healthier food items—and signify the programme’s immediate and mid-term effectiveness in tackling the barrier of knowledge or lack thereof.

Confidence in cookery practice has been shown to be an impactful contributing factor to dietary consumption patterns [27]. For that reason, it is important to enhance confidence in order to provide motivation to practice positive cookery skills [10]. The programme demonstrates effectiveness in improving and sustaining cookery confidence levels which is consistent with earlier studies [10,16,28,29,30]. Increasing confidence is also important for social integration.

Impact on positive dietary behaviour change in the short-term can be challenging when implementing nutrition-based interventions without any underlying theoretical behavioural models [31,32]. In this study, a change in eating behaviour of the participants remained insignificant, most likely due to the short duration of the intervention and the lack of on-going positive reinforcement [31].

Another reason for this occurrence may be that most participants indicated mainly positive behaviours at the start of the programme, which were sustained through the programme—eating breakfast usually/always; eating meals at regular times usually; and sometimes eating snacks. This could, however, also indicate self-reporting bias [33]. While behavioural interventions are geared to help support dietary change through a wide range of required activities [34], one report suggests that the lack of reliable cookery evaluations is highly responsible for the vague link between dietary behaviours and cooking skills influences [9]. While the quantitative evaluation showed no immediate improvements in the consumption of specific food groups—salad and oily fish—from baseline to post-intervention, the results at follow-up revealed that participants were consuming more healthy food items. This was supported by the qualitative findings which indicate a shift towards healthier eating behaviours and practices. These findings of improved healthy eating patterns are consistent with other cooking studies [10,16,21,29]. Similarly, in the current study at follow-up interviews, 36% acknowledged eating fewer snacks during the day due to consuming proper meals, with some revealing that their usual snack had been exchanged for a healthier option, such as a piece of fruit.

Since only 27% of the 62 baseline-post-intervention evaluation participants completed all three questionnaires, which is a much lower response rate than others observed in the literature—47%, 43%, 47%, and 58%, respectively [10,16,29,30]—the quantitative results must be interpreted with caution. The low response rate for questionnaire completion during the follow-up evaluation might have been attributed to a seasonal effect because it was conducted in the summer when many people are away from home. A further reason might be the lack of interest of participants to engage with questionnaires due to low literacy or being vulnerable, or lengthy questionnaires. This suggests it might be necessary to explore other routes for improving participation for future evaluations, such as collecting responses via telephone interviews which had a higher response rate (54%), and fell reasonably in between other indicated interview response rate values in the literature [16,29].

Additionally, while there may be statistical inconsistencies noticed between similar constructs from Table 2 and Table 6 (i.e., planning meals before shopping and using leftovers), the context of these two questions are different as one measures confidence (not at all confident to very confident) in performing an action, while the other measures performing the action (never to always) itself. Hence, a participant may report being much more confident in planning a meal before shopping post programme (significant change), but has not fully initiated the practice yet, so the action remains relatively similar (non-significant change). Another explanation to this inconsistency could be how each question was interpreted by the participants, suggesting that changes to question wording is needed for any future evaluation testing similar outcomes.

A main limitation of this study is the lack of a randomized element and a true control group. The suitability of a randomized control trial design for this population in a community-based setting is usually challenging due to feasibility issues, yet Halligan et al. conducted an 80 participant randomized control trial pilot study which found that community-based cooking programmes can be feasible when they adopt community recruitment strategies and use a waiting list control group randomization design [35]. Another limitation is that we did not use a validated dietary assessment tool to obtain dietary behavioral data thus a recommendation for future evaluations testing similar outcomes is to collect data through 24-h recall or food diary questionnaires [35]. The questionnaires used in the current study were considered to be a slight burden and barrier to starting the first session on time because of their length and difficulty. These potential problems may be reflected in the number of questionnaires that were returned partially completed at baseline (17.9%), post-intervention (8.8%), and follow-up (26.1%). Nonetheless, the overall quantitative results need to be interpreted with caution, as only 14% of the original universal course participants completed all three-evaluation elements, which is similar to the 10% response rate found in a 1-year long-term study [16], and lower than the 24% [29] and 31% [10] response rates found in 6-month long-term studies.

Sampling and response biases were perceived as limitations in the recruitment and evaluation phases of the programme. Many stated that they attended (or were recruited to) the programme because of a true interest in learning how to cook. This may signify a sampling or selection bias, as those who are interested are “naturally drawn” to the course [11], and those who are not remain absent—an important group of the intended survey population. Additionally, as the sample of 17 was not a true representation of the 62 baseline and post-intervention samples, participation/ attrition bias can be implied as a source of prejudice and a limitation for analysis and robustness of the findings. While many factors and reasons may have led to the differences in response rates at the three time points, such as the varying demographics between the two samples (e.g., gender and age), the complete control of participatory evaluation retention is impossible to maintain. Using self-reported questionnaires as an evaluation tool can lead to response bias, as the participants may feel the desire to over or under-report in order to impress or inform the evaluators what they want to hear [10,33]. A further limitation was the short follow-up time which does not allow direct comparisons with other studies in which 6 months is a common time for follow up. Nevertheless, our findings reflect mid-term sustainability of several outcomes and were confirmed by qualitative findings, but a longer term evaluation is needed.

## 5. Conclusions

The study is a small sample analysis and it is difficult to generalize the outcomes, however, the Eat Better Feel Better cooking programme was effective in reducing the barriers of cost and waste, in managing time better, and in increasing confidence and knowledge in the planning, purchasing, preparation, and cooking of healthy meals. Immediate outcomes indicate that the participants benefited from the programme as evidenced by: an increased shift towards healthier eating habits; decreased waste production and food spending; increased mindfulness in making healthy food choices; and using time more effectively and efficiently in the cookery process. Most of these improvements were sustained three to four months after programme implementation. The qualitative responses demonstrated an extended reach to family members and the value of providing opportunities to socialize. Overall, the programme has proven to be successful in positively impacting participants’ cookery practices and skills, in both the short and mid-term; and adds to the body of evidence supporting the benefits of cookery programmes in encouraging a healthy diet by tackling the barriers of time, cost, waste, and knowledge. Our study had limitations because of lack of a control group and small sample size at follow-up. Nevertheless we have conducted a comprehensive evaluation of effectiveness which allows us to propose this as a suitable programme to be incorporated into practical recommendations to align with healthy eating policies for obesity prevention in the Scottish context. This can have positive implications for practitioners but also for population health.

## Figures and Tables

**Table 1:** Participant demographics of the Eat Better Feel Better 6-week cooking skills programme.

Demographics	Baseline and Post-Intervention		3–4 Months Follow-Up		*p*-Value *
	*n*	%	*n*	%	
Sample size	62	100.0	17	100.0	
Completed Evaluations					
Yes	62	53.0	17	27.4	
No	55	47.0	45	72.6	
No. of Sessions Attended					0.152
3–4	10	16.0	1	5.9	
5–6	32	51.7	10	58.8	
Missing	20	32.3	6	35.3	
Gender					0.754
Female	42	67.7	11	64.7	
Male	20	32.3	6	35.3	
Age (years)					0.016 *
≤16	7	11.3	0	0.0	
17–24	4	6.5	1	5.9	
25–34	8	12.9	0	0.0	
35–44	8	12.9	2	11.8	
≥45	35	56.5	14	82.4	
Ethnic Background					0.276
Scottish	59	95.2	16	94.1	
Other British	1	1.6	0	0.0	
Other White background	1	1.6	1	5.9	
Pakistani	1	1.6	0	0.0	
No. of Household Members					0.042 *
1–2	37	59.7	14	82.4	
3–4	15	25.2	1	5.9	
5–7	3	4.8	0	0.0	
Missing	7	11.3	2	11.8	
Location					0.043 *
Glasgow City	18	29.0	2	11.8	
Inverclyde	27	43.5	6	35.3	
Renfrewshire	15	24.2	8	47.1	
East Renfrewshire	2	3.2	1	5.9	
SIMD ^1^					0.499
Quintile 1	36	58.1	9	52.9	
Quintile 2	11	17.7	4	23.5	
Quintile 3	3	4.8	2	11.8	
Quintile 4	0	0.0	0	0.0	
Quintile 5	3	4.8	1	5.9	
Missing	9	14.5	1	5.9	

Notes: ^1^ SIMD, Scottish Index of Multiple Deprivation, * *p*-value significance accepted at *p* < 0.05 using the Chi-Square Test—Likelihood Ratio for comparisons between baseline/post-intervention and 3–4 months follow-up.

**Table 2:** Median values for food preparation, cooking, and eating practices at baseline, post-intervention (*n* = 62), and 3–4 months follow up (*n* = 17).

Cooking and Eating Practices	Baseline and Post Intervention Completers ^1^			Baseline, Post, and Follow Up Completers ^2^			
	Baseline	Post	Post-Baseline	Baseline	Post	Follow Up	Follow Up-Baseline
	Median ^3^ (P25, P75) ^4^	Median (P25, P75)	*p-*Value *	Median (P25, P75)	Median (P25, P75)	Median (P25, P75)	*p*-Value
I think about how I can save time cooking	3 (2, 4)	3 (2, 4)	0.105	3 (2, 3)	3 (3, 4)	3 (2, 3)	0.669
I think it is time consuming using raw ingredients for cooking	3 (2, 4)	3 (2, 3)	0.123	3 (1, 4)	2 (1, 3)	2 (1, 3)	0.227
I plan what to cook before shopping	3 (2, 3)	3 (2, 4)	0.068	3 (2, 3)	3 (2, 4)	4 (3, 4)	0.008 *
I look for special offers on food when I shop	4 (3, 5)	4 (3, 5)	0.619	4 (3, 5)	4 (3, 4.5)	4 (3, 4)	0.729
I cook in bulk	2 (1, 3)	3 (1, 3)	0.039 *	2.5 (1, 3)	3 (1.5, 4)	3 (3, 3.8)	0.038 *
I throw away leftover food	3 (2, 4)	3 (2, 4)	0.394	3 (2, 3.7)	2 (2, 3.8)	2 (1, 3)	0.017 *
I eat breakfast	4 (3, 5)	5 (3, 5)	0.156	5 (3.5, 5)	4.5 (3, 5)	5 (2.5, 5)	0.521
I have snacks in between meals	3 (3, 4)	3 (2, 4)	0.226	3 (3, 3.8)	3 (2, 4)	3 (2, 4)	0.317
I eat meals at regular times	4 (3, 5)	4 (3, 5)	0.284	4 (4, 4.8)	4 (4, 4.5)	4 (4, 4.5)	1.000

Notes: ^1^ N ranged between 57–62; ^2^ N for follow up was 17; ^3^ Likert scale values from 1 to 5: 1 = never; 2 = rarely; 3 = sometimes; 4 = usually; 5 = always; ^4^ P25, 25th percentile, P75, 75th percentile; * *p*-value significance accepted at *p* < 0.05 using post hoc Wilcoxon Signed-Rank Test.

**Table 3:** Median values for the frequency of consumption of certain foods at baseline and post-intervention (*n* = 62) and 3–4 months follow-up (*n* = 17).

Selected Food Items	Baseline and Post-Intervention Completers ^1^			Baseline, Post-Intervention and Follow Up Completers ^2^			
	Baseline	Post	Post-Baseline	Baseline	Post	Follow Up	Follow Up-Baseline
	Median ^3^ (P25, P75) ^4^	Median (P25, P75)	*p*-Value *	Median (P25, P75)	Median (P25, P75)	Median (P25, P75)	*p*-Value
Leftover foods	2 (1, 3)	2 (1, 3)	0.022 *	2 (1.25, 3)	2 (2, 4)	3 (1, 4)	0.132
Pre-prepared foods	3 (2, 4)	3 (2, 4)	0.032 *	3 (2, 4)	3 (2, 4)	2 (2, 4)	0.058
Salad	3 (2, 4)	3 (2, 4)	0.736	3 (2, 3)	3 (2, 3.5)	4 (3, 5)	0.024 *
Oily fish	2 (1, 3)	2 (1, 3)	0.499	2 (1, 3.5)	3 (1, 4)	4 (2, 4)	0.039 *

Notes: ^1^ N ranged between 59–62; ^2^ N for follow up was 17 (range 15–17); ^3^ Likert scale values from 1 to 7: 1 = never, 2 = less than once a week, 3 = once a week, 4 = 2–4 times a week, 5 = 5–6 times a week, 7 = more than once a day; ^4^ P25, 25th percentile, P75, 75th percentile; * *p*-value significance accepted at *p* < 0.05 using post hoc Wilcoxon Signed-Rank Test.

**Table 4:** Correct and incorrect responses to the sugar content of common breakfast cereals at baseline and post-intervention (*n* = 62) and 3–4 months follow-up (*n* = 17).

Selected Cereals	Baseline ^1^			Post-Intervention ^1^			Baseline ^2^			Post-Intervention ^2^			Follow-Up ^2^		
	C	IN	NR	C	IN	NR	C	IN	NR	C	IN	NR	C	IN	NR
Cornflakes ^†^	58	31	11	50	40	10	71	19	12	41	47	12	71	6	23
Plain Porridge ^†^	89	5	6	84	8	8	94	0	6	88	6	6	76	0	23
Coco Pops ^†^	84	5	11	87	2	11	82	6	12	88	0	12	71	6	24
Crunchy Nut Cornflakes ^†^	63	26	11	68	23	10	71	18	12	82	6	12	71	6	24
Rice Krispies ^†^	53	36	11	60	31	10	53	35	12	59	29	12	35	41	24
Weetabix ^†^	71	19	10	74	18	8	71	23	6	82	12	6	59	18	24

Notes: ^1^ N for baseline and post-intervention was 62; ^2^ N for follow up was 17; C, correct percentage; IN, incorrect percentage; NR, no response. ^†^ Based on a scale value: low sugar, medium sugar, and high sugar content, where: cornflakes = medium sugar; plain porridge = low sugar; coco pops = high sugar; crunchy nut cornflakes = high sugar; rice krispies = medium sugar; Weetabix = low sugar. No statistically significant changes between baseline and post-intervention for all cereals.

**Table 5:** Correct and incorrect responses to the fat content of common food products at baseline and post-intervention (*n* = 62) and 3–4 month follow-up (*n* = 17).

Selected Food Items	Baseline ^1^			Post-Intervention ^1^			Baseline ^2^			Post-Intervention ^2^			Follow-Up ^2^		
	C	IN	NR	C	IN	NR	C	IN	NR	C	IN	NR	C	IN	NR
Plain Scones ^†^	57	37	7	69	24	7	76	18	6	88	6	6	65	12	23
Fresh Fruit ^†^	84	11	5	85	10	5	94	6	0	88	6	6	77	0	23
Standard Bag of Crisps ^†^	66	27	7	69	26	5	82	12	6	88	6	6	59	18	23
Sausage Roll ^†,^*	74	19	6	90	5	5	88	6	6	82	12	6	77	0	23
Baked Crisps ^†^	48	45	6	61	34	5	65	29	6	88	6	6	41	35	23
Vegetable Soup ^†^	77	16	6	82	15	3	82	12	6	94	6	0	71	6	23

Notes: ^1^ N for baseline and post-intervention was 62; ^2^ N for follow up was 17; C, correct percentage; IN, incorrect percentage; NR, no response. ^†^ Based on a scale value: low fat, medium fat, and high fat content, where: plain scones = medium fat; fresh fruit = low fat; standard bag of crisps = high fat; sausage roll = high fat; baked crisps = medium fat; vegetable soup = low fat. * Statistically significant difference between baseline^1^ and post-intervention^1^ for correct knowledge of sausage roll fat content (*p* = 0.011).

**Table 6:** Confidence ratings for preparation and cooking practices at baseline and post intervention (*n* = 62) and 3–4 month follow-up (*n* = 17).

Food Preparation Practices	Baseline and Post Intervention Completers ^1^			Baseline, Post, and Follow Up Completers ^2^			
	Baseline	Post	Post-Baseline	Baseline	Post	Follow Up	Follow Up-Baseline
	Median ^3^ (P25, P75) ^4^	Median (P25, P75)	*p*-Value	Median (P25, P75)	Median (P25, P75)	Median (P25, P75)	*p*-Value
Cooking using raw ingredients	4 (3, 6)	6 (3, 7)	0.003 *	5 (3, 6.5)	6 (2, 7)	7 (5, 7)	0.160
Following a simple recipe	5 (4, 6)	6 (4, 7)	0.003 *	5 (3.25, 6)	6 (4.5, 7)	7 (5, 7)	0.038 *
Planning meals before shopping	4 (2, 5)	5 (3, 6)	<0.05 *	4 (2, 5.5)	6 (3.5, 6)	5 (3, 7)	0.030 *
Shopping on a budget	4 (3, 6)	5 (4, 6)	0.044 *	4 (2, 5.5)	5 (4.5, 6)	6 (4, 7)	0.037 *
Shopping for healthier food	4 (3, 6)	5 (4, 6)	0.007 *	4 (2, 5)	5 (4, 6)	5 (4, 7)	0.008 *
Cooking new foods	3 (2, 5)	5 (3, 6)	<0.05 *	2 (1, 4)	5 (3.5, 6)	4 (4, 6)	0.002 *
Cooking healthier foods	4 (3, 5)	5 (3, 6)	0.001 *	4 (2, 5)	6 (4, 6)	5 (5, 7)	0.006 *
Storing food safely	5 (4, 7)	6 (4, 7)	0.002 *	4 (3.5, 7)	6 (4.5, 7)	7 (5, 7)	0.011 *
Using leftovers for other meals	4 (2, 5)	5 (3, 6)	0.002 *	3.5 (2, 5)	5 (4, 7)	5 (3, 7)	0.040 *
Cooking whole raw chicken	5 (2, 7)	6 (3, 7)	0.021 *	5 (2.5, 7)	6 (5, 7)	6 (5, 7)	0.087
Reading food labels	4 (2, 6)	5 (4, 7)	0.000 *	3 (2.5, 5)	5 (4, 6)	6 (4, 7)	0.001 *
Food hygiene	6 (5, 7)	6 (5, 7)	0.624	5 (4, 6.5)	6 (5.5, 7)	7 (5, 7)	0.043 *

Notes: ^1^ N ranged between 57 and 62; ^2^ N for follow up was 17; ^3^ Likert scale values from 1 to 7: 1 = not at all confident, 7 = very confident. ^4^ P25, 25th percentile, P75, 75th percentile; * *p*-value significance accepted at *p* < 0.05 using post hoc Wilcoxon Signed-Rank Text.

## Supplementary Materials

The following are available online at www.mdpi.com/1660-4601/14/4/380/s1, Supplementary Material 1 Baseline, Post-Intervention and Follow-up Questionnaires, Supplementary Material 2 Follow-up Telephone Interview, Supplementary material 3 Table S1: Median values for food preparation, cooking and eating practices at baseline and post-intervention stratified by attendance, Table S2: Confidence ratings for preparation and cooking practices at baseline and post intervention stratified by attendance.

## Abbreviations

α	alpha
ANOVA	Analysis of Variance
MVLS	Medical, Veterinary and Life Sciences
NHSGGC	National Health Service in Greater Glasgow and Clyde
*p*	*p*-value
SIMD	Scottish Index of Multiple Deprivation
UK	United Kingdom
£	pound currency
