# Supplementary material for: Evaluation of the “Eat Better Feel Better” Cooking Programme to Tackle Barriers to Healthy Eating
Source: Int J Environ Res Public Health. 2017 Apr 4;14(4):380. doi: 10.3390/ijerph14040380 (PMC5409581; doi:10.3390/ijerph14040380)
Supplement: Supplementary file 1 [file ijerph-14-00380-s001.zip › ijerph-165970/Supplemmentary material 1_Evaluation questionnaires Baseline Post Follow up.pdf]

#

NHSGGC Community-based Cooking Skills Programme
Baseline Questionnaire

Identification number: ___________________

1. **For your main meals, what kind of cooking do you mainly do at the moment? (Please tick as many boxes as apply)**
   - Don't cook at all ☐
   - Put ready meals in microwave or oven ☐
   - Put together ready-made ingredients (sauce jars) to make a meal ☐
   - Prepare meals from scratch (using raw ingredients) ☐

2. **On a scale of 1 (never) to 5 (always) please tick which best currently describes you, as shown in the example below**: #

	1 Never	2 Rarely	3 Some-times	4 Usually	5 Always
Example: I buy food in farmer's markets?	☞				
When I shop, I think about how I can save time cooking					
I think it is time consuming to use raw ingredients when cooking					
I plan what to cook before I go shopping					
I look for special offers on foods when I shop					
I cook in bulk (to save portions for other meals)					
I throw away leftover food					
I eat breakfast					
I have snacks in between meals					
I eat meals at regular times					

3. **Do you know why it is important to eat a balanced diet?**
   - Yes ☐
   - No ☐ (Please go to Question 5)

4. **Can you tell us why you think a balanced diet is important?**

#
#
##

# #

5. Which breakfast cereals do you think are low, medium and high in sugar from the list below? Please indicate with 'L' for low, 'M' for medium and 'H' for High in the boxes provided.

Cornflakes ☐
Plain Porridge ☐
Coco Pops ☐
Crunchy Nut cornflakes ☐
Rice Crispies ☐
Weetabix ☐

6. Which foods do you think are low, medium and high in fat from the list below? Please indicate with 'L' for low, 'M' for medium and 'H' for High in the boxes provided.

Plain scone ☐
Fresh fruit ☐
Standard bag of crisps ☐
Sausage roll ☐
Baked crisps ☐
Vegetable soup ☐

7. Do you understand correct portion sizes?

Yes ☐
No ☐
I'm not sure ☐

8. What foods do you think you eat too much of for a balanced diet?

#
#
#

9. What foods do you think you eat too little of for a balanced diet?

#
#
#

10. Roughly, how much money per week do you spend on food? #

£ per week:

11. Roughly, how much money per week do you spend on takeaways/fast food?

£ per week:

#

12. In a normal week, how often do you eat the following foods? (Please tick just one box per food as in the example)

	Never	Less than once a week	Once a week	2-4 times a week	5-6 times a week	Once a day	More than once a day
Example: cakes			✓				
Re-used foods (e.g. leftovers)							
Ready meals (e.g. frozen pizza, microwave meals)							
Salad (e.g. using fresh veg)							
Oily fish (e.g. salmon or sardines)							

13. On a scale of 1 (not confident at all) to 7 (very confident) please tick which number best shows how confident you feel about the following: (please tick one box)

	Not at all confident 1	2	3	4	5	6	Very confident 7
Example: Baking							✓
Cooking using raw ingredients							
Following a simple recipe							
Planning meals before shopping							
Shopping for food on a budget							
Shopping for healthier food to eat							
Cooking new foods							
Cooking healthier foods							
Storing food safely							
Using food leftovers to cook other meals							
Cooking whole raw chicken from scratch							
Reading food labels							
Food Hygiene							

14. When you buy food, do you look at any of this information on the label?

Calories: Yes☐ No☐#

Fat: Yes ☐ No☐#

Sugars: Yes ☐ No☐#

Sodium/salt:
Yes☐ No☐#

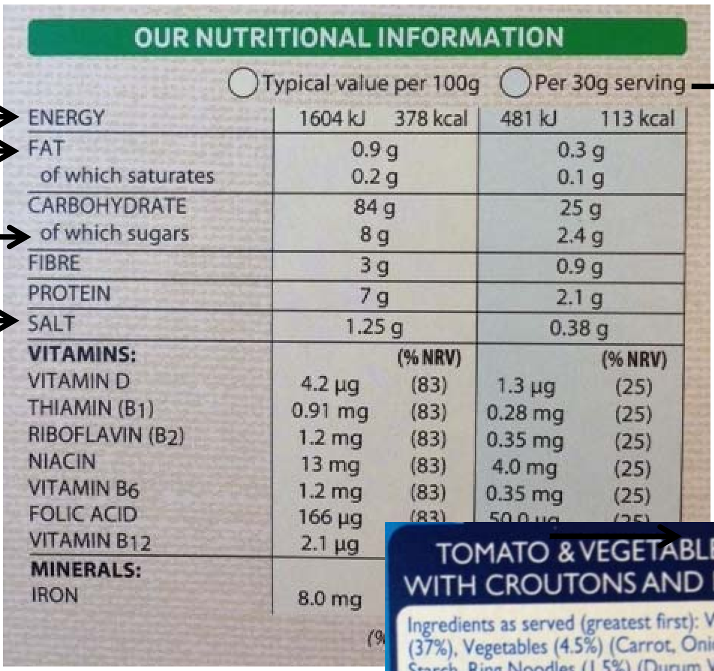

Portion size:
Yes ☐ No☐

Ingredient list: Yes☐ No☐#

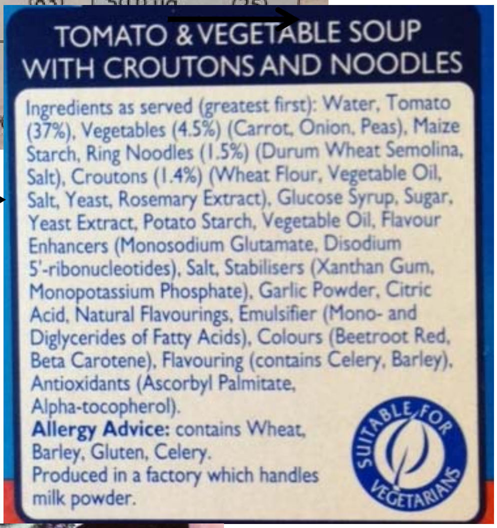

Use by date: Yes☐ No☐#

Total Price:
Yes ☐ No☐#

Price per g or kilogram:
Yes☐ No☐#

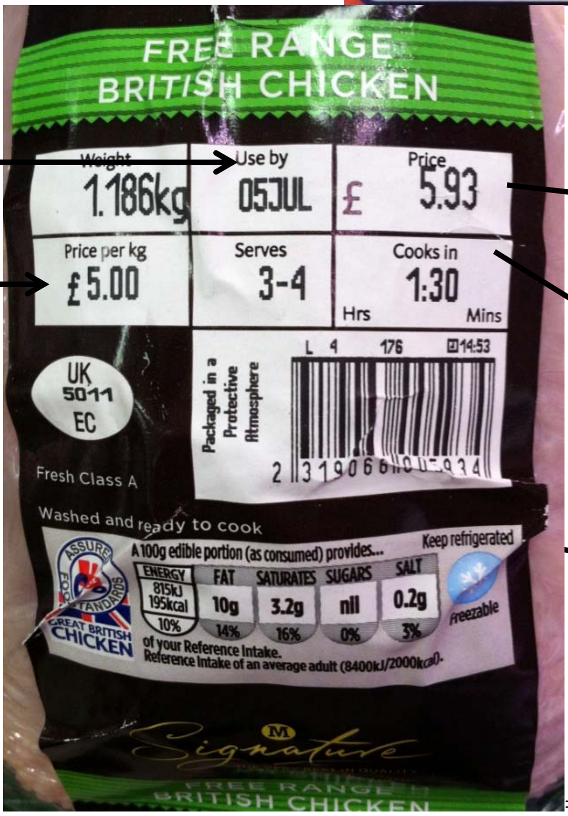

#

Cooking tips:
Yes ☐ No☐#

Storage tips:
Yes ☐ No☐#

# #

Details about yourself............

What age are you?

- 16 or under ☐
- 17-24 ☐
- 25-34 ☐
- 35-44 ☐
- 45 or over ☐

How would you describe your ethnic background? (Please tick one box)

White
- Scottish ☐
- Other British ☐
- Any other White background ☐

Asian, Asian Scottish or Asian British
- Indian ☐
- Pakistani ☐
- Bangladeshi ☐
- Chinese ☐
- Any other Asian background ☐

Black, Black Scottish or Black British
- Caribbean ☐
- African ☐
- Any other Black background ☐

Mixed
- Any mixed background ☐

Any other ethnic background (specify) ☐

Prefer not to say ☐

How many family members live in your household? ______

Are you male/female? (Please circle)

What is your postcode? ____________

I AGREE to my details being stored on a confidential data base and to information being shared between NHS Greater Glasgow and Clyde and approved partner organisations involved in this programme.	Yes ☐ No ☐
I AGREE to NHS Greater Glasgow and Clyde Health Improvement and/or the University of Glasgow contacting me to invite me to take part in an evaluation of the programme.<br><br>If yes, can you please provide a contact name and telephone number below:<br><br>Name:<br><br>Telephone Number:	Yes ☐ No ☐

Thank you for taking the time to complete this questionnaire

#

NHSGGC Community-based Cooking Skills Programme
Post-Intervention Questionnaire

Identification number: ___________________

1. For your main meals, what kind of cooking do you mainly do at the moment? (Please tick as many boxes as apply)

- Don't cook at all ☐
- Put ready meals in microwave or oven ☐
- Put together ready-made ingredients (sauce jars) to make a meal ☐
- Prepare meals from scratch (using raw ingredients) ☐

2. On a scale of 1 (never) to 5 (always) please tick which best currently describes you, as shown in the example below: #

	1 Never	2 Rarely	3 Some-times	4 Usually	5 Always
Example: I buy food in farmer's markets?	☞				
When I shop, I think about how I can save time cooking					
I think it is time consuming to use raw ingredients when cooking					
I plan what to cook before I go shopping					
I look for special offers on foods when I shop					
I cook in bulk (to save portions for other meals)					
I throw away leftover food					
I eat breakfast					
I have snacks in between meals					
I eat meals at regular times					

3. Do you know why it is important to eat a balanced diet?

Yes ☐

No ☐ (Please go to Question 5)

4. Can you tell us why you think a balanced diet is important?

#
#
##

# #

5. Which breakfast cereals do you think are low, medium and high in sugar from the list below? Please indicate with 'L' for low, 'M' for medium and 'H' for High in the boxes provided.

Cornflakes ☐
Plain Porridge ☐
Coco Pops ☐
Crunchy Nut cornflakes ☐
Rice Crispies ☐
Weetabix ☐

6. Which foods do you think are low, medium and high in fat from the list below? Please indicate with 'L' for low, 'M' for medium and 'H' for High in the boxes provided.

Plain scone ☐
Fresh fruit ☐
Standard bag of crisps ☐
Sausage roll ☐
Baked crisps ☐
Vegetable soup ☐

7. Do you understand correct portion sizes?

Yes ☐
No ☐
I'm not sure ☐

8. What foods do you think you eat too much of for a balanced diet?

#
#
#

9. What foods do you think you eat too little of for a balanced diet?

#
#
#

10. Roughly, how much money per week do you spend on food? #

£ per week:

11. Roughly, how much money per week do you spend on takeaways/fast food?

#

£ per week:

#

12. In a normal week, how often do you eat the following foods? (Please tick just one box per food as in the example)

	Never	Less than once a week	Once a week	2-4 times a week	5-6 times a week	Once a day	More than once a day
Example: cakes			✓				
Re-used foods (e.g. leftovers)							
Ready meals (e.g. frozen pizza, microwave meals)							
Salad (e.g. using fresh veg)							
Oily fish (e.g. salmon or sardines)							

. On a scale of 1 (not confident at all) to 7 (very confident) please tick which number best shows how confident you feel about the following: (please tick one box)

	Not at all confident 1	2	3	4	5	6	Very confident 7
Example: Baking							✓
Cooking using raw ingredients							
Following a simple recipe							
Planning meals before shopping							
Shopping for food on a budget							
Shopping for healthier food to eat							
Cooking new foods							
Cooking healthier foods							
Storing food safely							
Using food leftovers to cook other meals							
Cooking whole raw chicken from scratch							
Reading food labels							
Food Hygiene							

14. When you buy food, do you look at any of this information on the label?

Calories: Yes☐ No☐#

Fat: Yes ☐ No☐#

Sugars: Yes ☐ No☐#

Sodium/salt:
Yes☐ No☐#

Portion size:
Yes ☐ No☐

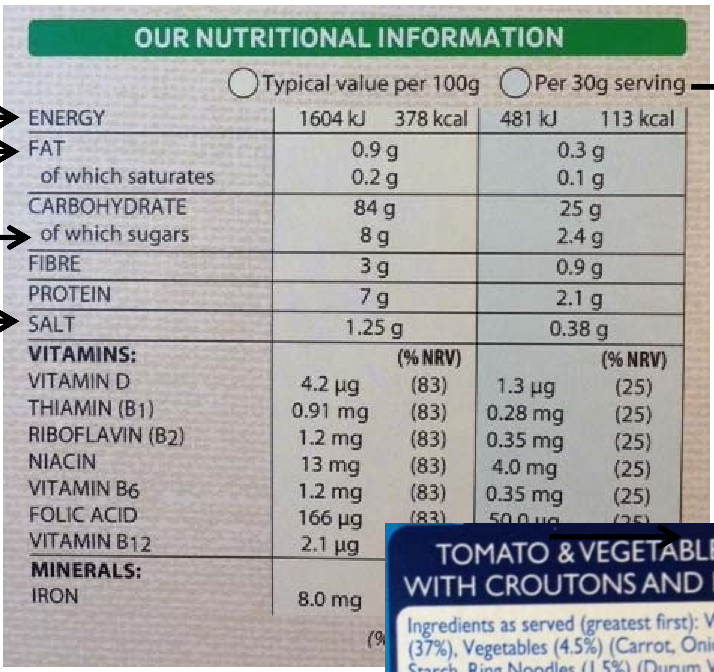

Ingredient list: Yes☐ No☐#

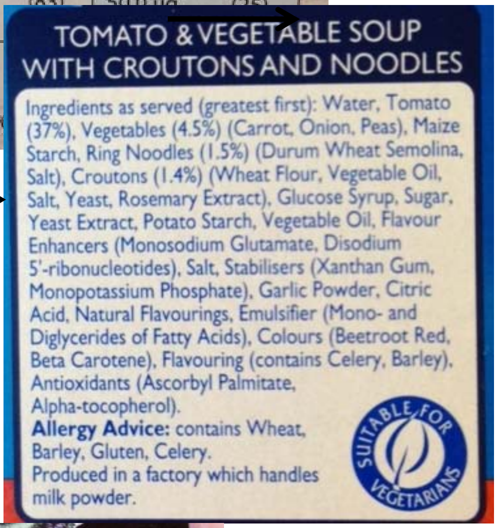

Use by date: Yes☐ No☐#

Price per g or kilogram:
Yes☐ No☐#

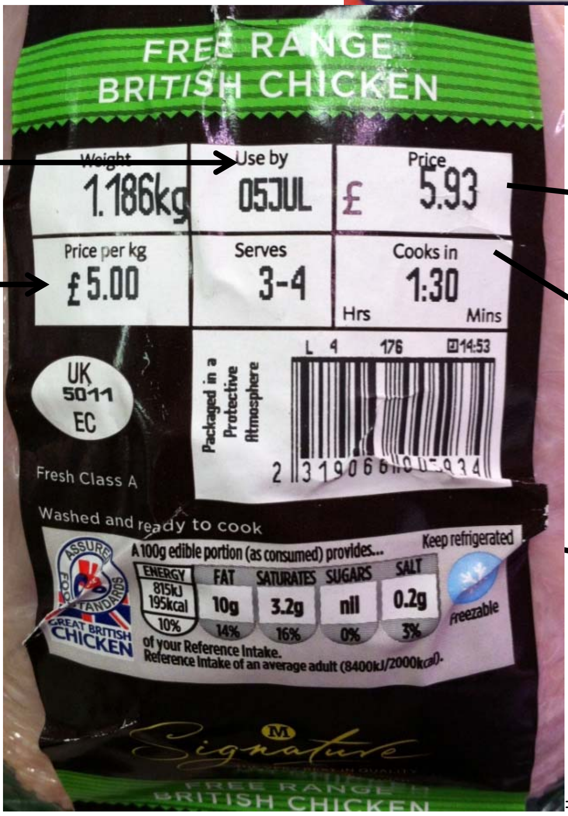

#

Total Price:
Yes ☐ No☐#

Cooking tips:
Yes ☐ No☐#

Storage tips:
Yes ☐ No☐#

Thank you for taking the time to complete this questionnaire!

# #

15. What did you enjoy about the course?

16. What didn't enjoy about the course?

17. What two things have you started to do at home as a result of attending this course?

1.

2.

18. Was there anything you struggled with or found difficult <u>during</u> the course?

1.

2.

19. Was there anything from the course you tried at home that was difficult or you could not do?

Yes ☐ No ☐

If yes, please explain what the challenges were?

20. Is there anything else you would like to tell us on how the course helped you?

Yes ☐ No ☐

If yes, please explain below:

Thank you for taking the time to complete this questionnaire

University of Glasgow | College of Medical, Veterinary & Life Sciences #

#

NHSGGC Community-based Cooking Skills Programme
Follow-up Questionnaire

Identification number: ___________________

1. For your main meals, what kind of cooking do you mainly do at the moment? (Please tick as many boxes as apply)

Don't cook at all ☐
Put ready meals in microwave or oven ☐
Put together ready-made ingredients (sauce jars) to make a meal ☐
Prepare meals from scratch (using raw ingredients) ☐

2. On a scale of 1 (never) to 5 (always) please tick which best currently describes you, as shown in the example below: #

	1 Never	2 Rarely	3 Some-times	4 Usually	5 Always
Example: I buy food in farmer's markets?	☞				
When I shop, I think about how I can save time cooking					
I think it is time consuming to use raw ingredients when cooking					
I plan what to cook before I go shopping					
I look for special offers on foods when I shop					
I cook in bulk (to save portions for other meals)					
I throw away leftover food					
I eat breakfast					

3. Do you know why it is important to eat a balanced diet?

Yes ☐
No ☐ (Please go to Question 5)

4. Can you tell us why you think a balanced diet is important?

#
#
##

# #

5. Which breakfast cereals do you think are low, medium and high in sugar from the list below? Please indicate with 'L' for low, 'M' for medium and 'H' for High in the boxes provided.

Cornflakes ☐ Crunchy Nut cornflakes ☐

Plain Porridge ☐ Rice Crispies ☐

Coco Pops ☐ Weetabix ☐

6. Which foods do you think are low, medium and high in fat from the list below? Please indicate with 'L' for low, 'M' for medium and 'H' for High in the boxes provided.

Plain scone ☐ Sausage roll ☐

Fresh fruit ☐ Baked crisps ☐

Standard bag of crisps ☐ Vegetable soup ☐

7. Do you understand correct portion sizes?

Yes ☐
No ☐
I'm not sure ☐

8. What foods do you think you eat too much of for a balanced diet?

#
#
#

9. What foods do you think you eat too little of for a balanced diet?

#
#
#

10. Roughly, how much money per week do you spend on food? #

£ per week:

11. Roughly, how much money per week do you spend on takeaways/fast food?

£ per week:

#

12. In a normal week, how often do you eat the following foods? (Please tick just one box per food as in the example)

	Never	Less than once a week	Once a week	2-4 times a week	5-6 times a week	Once a day	More than once a day
Example: cakes			✓				
Re-used foods (e.g. leftovers)							
Ready meals (e.g. frozen pizza, microwave meals)							
Salad (e.g. using fresh veg)							
Oily fish (e.g. salmon or sardines)							

13. On a scale of 1 (not confident at all) to 7 (very confident) please tick which number best shows how confident you feel about the following: (please tick one box)

	Not at all confident 1	2	3	4	5	6	Very confident 7
Example: Baking							✓
Cooking using raw ingredients							
Following a simple recipe							
Planning meals before shopping							
Shopping for food on a budget							
Shopping for healthier food to eat							
Cooking new foods							
Cooking healthier foods							
Storing food safely							
Using food leftovers to cook other meals							
Cooking whole raw chicken from scratch							
Reading food labels							
Food Hygiene							

14. When you buy food, do you look at any of this information on the label?

Calories: Yes☐ No☐#

Fat: Yes ☐ No☐#

Sugars: Yes ☐ No☐#

Sodium/salt:
Yes☐ No☐#

Portion size:
Yes ☐ No☐

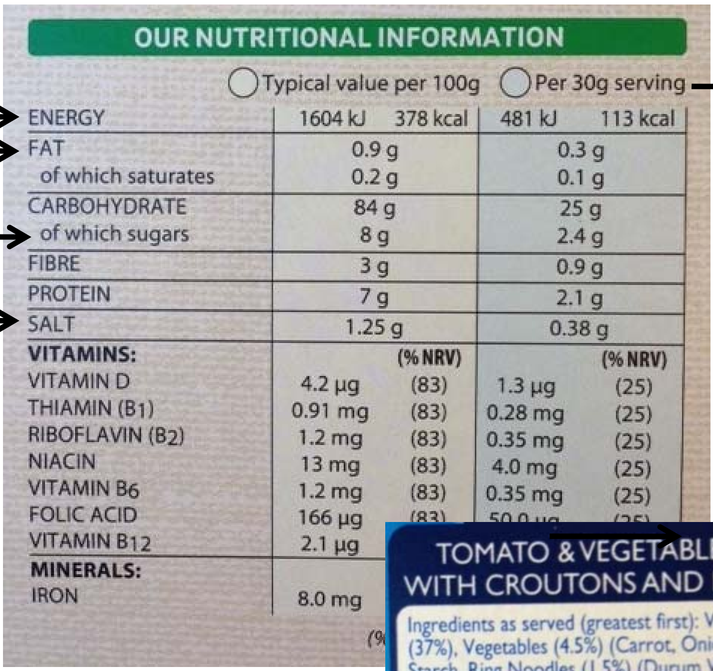

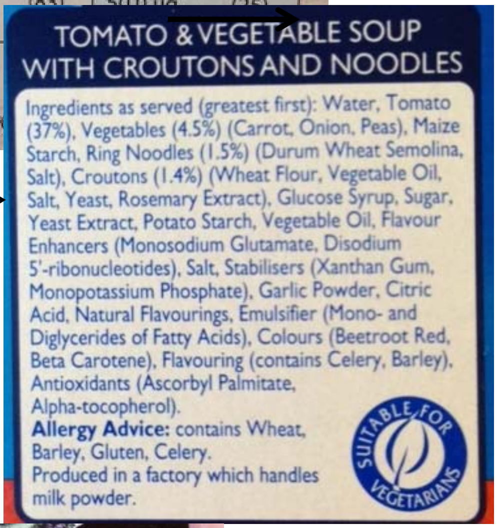

Ingredient list: Yes☐ No☐#

Use by date: Yes☐ No☐#

Price per g or kilogram:
Yes☐ No☐#

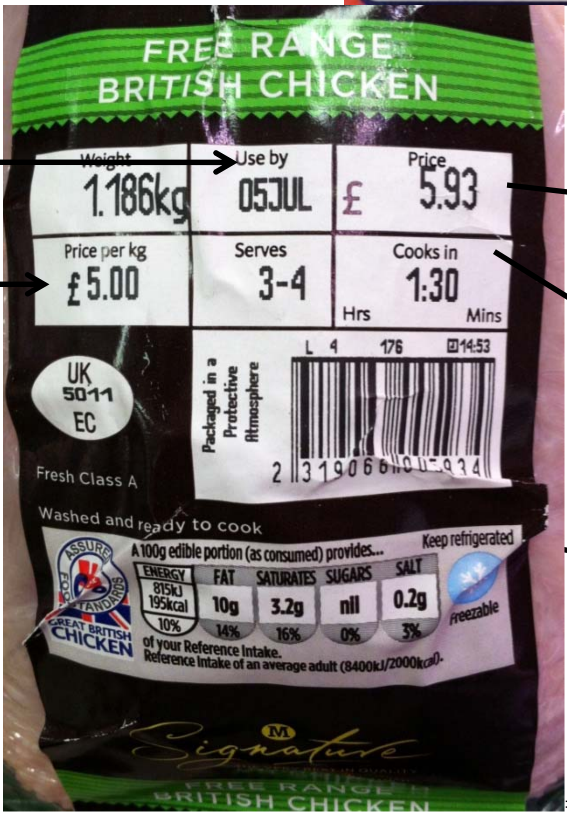

#

Total Price:
Yes ☐ No☐#

Cooking tips:
Yes ☐ No☐#

Storage tips:
Yes ☐ No☐#

Thank you for taking the time to complete this questionnaire!

# #

# #
