# Supplementary material for: Evaluation of the “Eat Better Feel Better” Cooking Programme to Tackle Barriers to Healthy Eating
Source: Int J Environ Res Public Health. 2017 Apr 4;14(4):380. doi: 10.3390/ijerph14040380 (PMC5409581; doi:10.3390/ijerph14040380)
Supplement: Supplementary file 1 [file ijerph-14-00380-s001.zip › ijerph-165970/Supplemmentary material 2_ Phone Interview Schedule.pdf]

University of Glasgow | College of Medical, Veterinary & Life Sciences#
#

EAT BETTER FEEL BETTER

NHS Greater Glasgow and Clyde

NHSGGC Community-based Cooking Skills Programme
Follow-up Questionnaire for Telephone Interview

Identification number: ___________________

1. What made you come along to the course?

2. What motivated you to stay to the end of the course?

3. What was the most important thing you learnt at the course?

4. Is there anything you have continued to do, since attending the course?

5. Was there anything you tried to do at home but do not do anymore?
   a. Yes ☐ No ☐
   b. If yes, what were they? And why did you stop?

6. Do you think this course was of help to you or your family?
   a. Yes ☐ No ☐
   b. If yes in which way?

7. Has your shopping changed?
   a. Yes ☐ No ☐
   b. If yes, how has it changed?
   c. If no, why do you think it has not changed?

8. Are you spending less money on food shopping?
   a. Why or why not?

9. Do you prepare more meals and snacks from raw ingredients?
   a. Yes ☐ No ☐
   b. If yes, what motivated you to do this?

10. For snacks, do you find yourself eating more, less or the same amount?
    a. More ☐ Less ☐ Same ☐
    b. Why do you think this is?

11. Do you waste less food?
    a. If yes, please explain in what way? E.g. types of foods?
    b. If no, what do you think are the main reasons?

12. Do you have any additional comments or suggestions on how to improve the course?

Thank you!
