# Supplementary material for: Evaluation of the “Eat Better Feel Better” Cooking Programme to Tackle Barriers to Healthy Eating
Source: Int J Environ Res Public Health. 2017 Apr 4;14(4):380. doi: 10.3390/ijerph14040380 (PMC5409581; doi:10.3390/ijerph14040380)
Supplement: Supplementary file 1 [file ijerph-14-00380-s001.zip › ijerph-165970/Supplemmentary material 3_Tables 1 & 2.pdf]

Supplementary Table 1. Median values for food preparation, cooking and eating practices at baseline and post-intervention stratified by attendance.

Cooking and Eating Practices	All participants[1]			Attendance 3-4 sessions[2]			Attendance 5-6 sessions[3]		
	Pre	Post		Pre	Post		Pre	Post	
	Median[4] (P25, P75)[5]	Median (P25, P75)	P value	Median (P25, P75)	Median (P25, P75)	P value	Median (P25, P75)	Median (P25, P75)	P value
I think about how I can save time when cooking	3 (2, 4)	3 (2, 4)	0.105	3 (2, 3)	3 (2, 4)	0.248	3 (2, 4)	3 (3, 4)	0.224
I think it is time consuming using raw ingredients when cooking	3 (2, 4)	3 (2, 3)	0.105	3 (2, 3)	2 (2, 2)	0.034*	3 (2, 4)	2 (1, 3)	0.249
I plan what to cook before shopping	3 (2, 3)	3 (2, 4)	0.068	3 (2, 4)	3 (1, 4)	0.705	3 (2, 4)	4 (3, 4)	0.006*
I look for special offers on food when I shop	4 (3, 5)	4 (3, 5)	0.619	4 (3, 4)	4 (2, 4)	0.558	5 (4, 5)	4 (3, 5)	0.322
I cook in bulk	2 (1, 3)	3 (1, 3)	0.039*	1 (1, 2)	3 (1, 3)	0.039*	3 (1, 3)	3 (2, 3)	0.191
I throw away leftover food	3 (2, 4)	3 (2, 4)	0.394	4 (4, 5)	4 (3, 4)	0.157	3 (2, 4)	3 (2, 4)	0.346
I eat breakfast	4 (3, 5)	5 (3, 5)	0.156	4 (1, 5)	5 (2, 5)	0.102	4 (3, 5)	5 (3, 5)	0.253
I have snacks in between meals	3 (3, 4)	3 (2, 4)	0.226	4 (3, 5)	3 (2, 5)	0.046	3 (3, 4)	3 (2, 4)	0.253
I eat meals at regular times	4 (3, 5)	4 (3, 5)	0.284	3 (2, 5)	4 (3, 5)	0.102	4 (4, 5)	4 (3, 5)	0.670

[1]N ranged between 57-62; [2]N for 3-4 sessions was 10, [3]N for 5-6 sessions ranged between 30-32; [4]Likert scale values from 1 to 5: 1=never; 2=rarely; 3=sometimes; 4=usually; 5=always; [5]P25, 25th percentile, P75, 75th percentile; *P-value significance accepted at $p<0.05$ using post hoc Wilcoxon Signed–Rank Test.

Supplementary Table 2. Confidence ratings for preparation and cooking practices at baseline and post intervention stratified by attendance

Food Preparation Practices	All participants[1]			Attendance 3-4 sessions[2]			Attendance 5-6 sessions[3]		
	Pre	Post		Pre	Post		Pre	Post	
	Median[4] (P25, P75)[5]	Median (P25, P75)	P value	Median (P25, P75)	Median (P25, P75)	P value	Median (P25, P75)	Median (P25, P75)	P value*
Cooking using raw ingredients	4 (3, 6)	6 (3, 7)	0.003*	3.5 (2.8, 2.3)	4.5 (3, 6.3)	0.139	5 (3.3, 7)	6 (4.3, 7)	0.009*
Following a simple recipe	5 (4, 6)	6 (4, 7)	0.003*	4 (1.8, 5.5)	5.5 (3.5, 7)	<0.05*	5.5 (4, 7)	7 (5.3, 7)	<0.05*
Planning meals before shopping	4 (2, 5)	5 (3, 6)	<0.05*	3 (1.8, .3)	4 (3, 6.5)	<0.05*	4 (3, 6)	6 (4, 6.8)	<0.05*
Shopping on a budget	4 (3, 6)	5 (4, 6)	0.044*	4.5 (4, 6.3)	4.5 (3, 5.3)	0.739	4.5 (3, 7)	6 (5, 7)	<0.05*
Shopping for healthier food	4 (3, 6)	5 (4, 6)	0.007*	4 (2.8, 6)	4.5 (4, 5)	0.365	4.5 (3.3, 6.8	6 (4, 6.8)	0.059
Cooking new foods	3 (2, 5)	5 (3, 6)	<0.05*	2 (1, 3.3)	3 (2, 4.3)	0.014*	4 (2, 5)	6 (5, 7)	0.001*
Cooking healthier foods	4 (3, 5)	5 (3, 6)	0.001*	3.5 (2.8, 5)	4 (2.8, 5)	0.680	4 (3.3, 5)	6 (5, 7)	0.001*
Storing food safely	5 (4, 7)	6 (4, 7)	0.002*	4 (4, 7)	6 (4.8, 7)	0.156	5 (4, 7)	6 (5.3, 7)	0.002*
Using leftovers for other meals	4 2, 5)	5 (3, 6)	0.002*	3 (2, 5.5)	4 (2, 5.3)	0.416	4 (2, 5)	5 (3, 7)	0.007*
Cooking whole raw chicken	5 (2, 7)	6 (3, 7)	0.021*	5 (2, 7)	6 (2, 7)	0.610	5 (2, 7)	7 (3.3, 7)	0.001*
Reading food labels	4 (2, 6)	5 (4, 7)	0.000*	2.5 (2, 4)	6 (2, 7)	0.027*	5 (3, 6)	6 (5, 7)	0.001*
Food hygiene	6 (5, 7)	6 (5, 7)	0.624	6.5 (4.8, 7)	6 (1.8, 7)	0.497	6 (5, 7)	7 (5, 7)	0.744

[1]N ranged between 57-62; [2]N for 3-4 sessions was 10, [3]N for 5-6 sessions ranged between 30-32; [4]Liker scale values from 1 to 7: 1=not at all confident, 7=very confident. [5]P25, 25th percentile, P75, 75th percentile; *P-value significance accepted at p<0.05 using post hoc Wilcoxon Signed–Rank Text.
